# Regulation of morphological differentiation in *S. coelicolor* by RNase III (AbsB) cleavage of mRNA encoding the AdpA transcription factor

**DOI:** 10.1111/j.1365-2958.2009.07023.x

**Published:** 2010-01-03

**Authors:** Weijing Xu, Jianqiang Huang, Richard Lin, Jing Shi, Stanley N Cohen

**Affiliations:** 1 Departments of Genetics, Stanford University School of MedicineStanford, CA 94305, USA; 2 Departments of Medicine, Stanford University School of MedicineStanford, CA 94305, USA

## Abstract

RNase III family enzymes, which are perhaps the most widely conserved of all ribonucleases, are known primarily for their role in the processing and maturation of small RNAs. The RNase III gene of *Streptomyces coelicolor*, which was discovered initially as a global regulator of antibiotic production in this developmentally complex bacterial species and named *absB* (*a*ntibiotic *b*io*s*ynthesis gene *B*), has subsequently also been found to modulate the cellular abundance of multiple messenger RNAs implicated in morphological differentiation. We report here that regulation of differentiation-related mRNAs by the *S. coelicolor* AbsB/RNase III enzyme occurs largely by ribonucleolytic cleavage of transcripts encoding the pleiotropic transcription factor, AdpA, and that AdpA and AbsB participate in a novel feedback-control loop that reciprocally regulates the cellular levels of both proteins. Our results reveal a previously unsuspected mechanism for global ribonuclease-mediated control of gene expression in streptomycetes.

## Introduction

*Streptomyces* species are prokaryotic soil-dwelling organisms that have a complex life cycle involving mycelial growth and spore formation. In addition to producing more than two-thirds of the antibiotics currently in clinical and veterinary use, *Streptomyces* are the source of multiple other types of pharmaceutically useful compounds (Challis and Hopwood, 2003). For more than 40 years, *Streptomyces coelicolor* has been widely employed as a model organism in studies of morphological development and antibiotic regulation in streptomycetes. These studies (reviewed in Bibb, 1996; Chater, 2006; Flardh and Buttner, 2009) plus more recent microarray-based investigations (Huang *et al.*, 2001; Huang *et al.*, 2005) have shown that antibiotic biosynthesis in *S. coelicolor* is controlled by a complex multifaceted regulatory network.

The *absB* gene was discovered in a screen for *S. coelicolor* mutants that are defective in antibiotic biosynthesis but which do not interfere with the morphological development associated with the production of antibiotics and other secondary metabolites in this micro-organism (Adamidis and Champness, 1992; Aceti and Champness, 1998). DNA sequence analysis suggested that the protein encoded by *absB* is a member of the RNase III family of endoribonucleases (Price *et al.*, 1999) – which commonly recognize double-strand segments of stem-loop structures and in bacteria carry out the processing of pre-rRNA, tRNA and polycistronic mRNA (Conrad and Rauhut, 2002; Drider and Condon, 2004). Cells containing a mis-sense point mutation in the *absB* gene were found to accumulate 30s rRNA precursors (Price *et al.*, 1999).

Subsequent studies have shown purified AbsB protein can carry out site-specific RNA cleavages *in vitro* (Chang *et al.*, 2005), and have provided direct evidence that the AbsB protein functions as a ribonuclease *in vivo* (Gravenbeek and Jones, 2008; Xu *et al.*, 2008). Additionally, microarray analysis of gene expression has shown that mutation of *absB* can dramatically and broadly affect the abundance of individual mRNAs in *S. coelicolor*, elevating the cellular levels of mRNAs implicated in sporulation while decreasing the abundance of mRNAs encoding activators of the biosynthesis of antibiotics and other secondary metabolites (Huang *et al.*, 2005).

The decrease in antibiotic biosynthesis-related transcripts (e.g. *redD*, *cdaR*, *actII-ORF4*, or *redZ*) observed in the *absB* mutant strain (Huang *et al.*, 2005), which is consistent with the defective antibiotic production phenotype associated with mutation of *absB* (Adamidis and Champness, 1992), implies that these *absB*-regulated transcripts are not substrates for the ribonucleolytic activity of AbsB/RNase III. In contrast, the increased abundance of sporulation gene transcripts (Huang *et al.*, 2005) raises the prospect that some or all of the sporulation gene transcripts elevated by *absB* mutation may be targets of ribonucleolytic digestion by the AbsB protein.

Here we report the discovery that AbsB/RNase III regulation of *S. coelicolor* genes implicated in morphological development is mediated by endoribonucleolytic cleavage of mRNA encoding AdpA, initially identified in *S. griseus* as an pleiotropic AraC/XylS family transcription factor (Ohnishi *et al.*, 2005). We further show that AbsB and AdpA participate in a post-transcriptional regulatory loop that modulates the cellular levels of the proteins encoded by both genes.

## Results

### Role of AdpA in AbsB-controlled gene expression

**Fig. 1:**
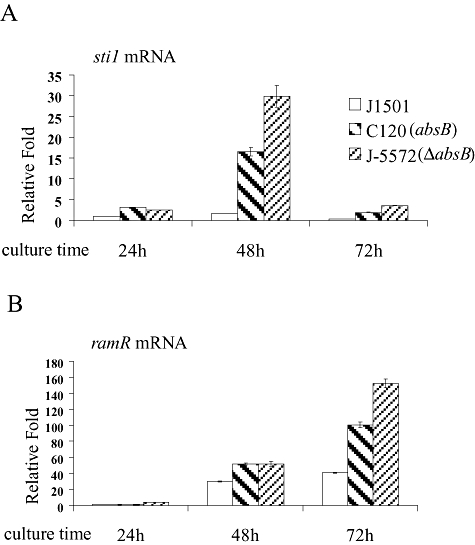
Quantitative RT-PCR analysis of abundance of (A) *sti1* and (B) *ramR* mRNA in *S. coelicolor* J1501 (morphologically wild type), C120 (*absB* point mutant) and J-5572 (Δ*absB*) strains. Mycelia were collected after 24 h, 48 h, 72 h of incubation, and RNA was extracted and purified using the methods described in *Experimental procedures*. Primer sequences used in quantitative PCR are listed in Table S3. The target cDNA was normalized internally to 16S rRNA. The abundance of the target cDNA was shown in the figure as the fold change relative to the cDNA abundance in J1501 at the 24 h time point. The bars indicate the Standard deviation from duplicate samples.

The transcript most dramatically elevated in *absB* mutant bacteria in microarray studies was *sti1* (SCO0762) (Huang *et al.*, 2005), which encodes a member of the subtilisin inhibitor (SSI) family of protease inhibitors found in multiple streptomycetes and which is known to be implicated in the formation of aerial mycelia and spores in *Streptomyces albogriseolus* (Taguchi *et al.*, 1995; Kato *et al.*, 2005). Direct assay in the *absB* point mutant strain C120, which replaces leucine by proline at position 120 of the *absB* open reading frame (Adamidis and Champness, 1992), showed increased sporulation (Fig. S1), and RT-PCR studies in the same strain as well as in an *absB* null mutant strain (J-5572) constructed in our lab (Fig. 1A) confirmed both the *absB* dependence and the temporally regulated elevation of *sti1* mRNA abundance relative to parental bacteria (J1501). This increase was most prominent 48 h after plating of spores – the time in the *S. coelicolor* life cycle associated with the formation of aerial mycelium. However, notwithstanding the dramatic increase in *sti1* transcript abundance observed in bacteria defective in *absB* (both in C120 and The *absB* null mutant strain J-5572), we did not detect cleavage of *sti1* transcripts by purified AbsB/RNase III protein (Fig. S2A) under conditions where the ribonuclease cleaved its own mRNA (Fig. S2B). These observations suggest that the regulation of at least *sti1* by AbsB is accomplished indirectly, rather than by AbsB/RNase III digestion of *sti1* transcripts. Potentially, such indirect regulation could be mediated by either an AbsB-targeted regulatory RNA or an AbsB-targeted mRNA encoding a transcription-activating protein.

*Sti1* is only one of many sporulation-gene-associated genes whose transcripts are elevated in *absB* mutant bacteria (Huang *et al.*, 2005). As an approach to identifying possible AbsB-targeted intermediates that may upregulate multiple sporulation-associated transcripts, we searched for features in common among AbsB-controlled genes. Expression of *sti1* (SCO0762) is TTA-dependent and is regulated in *S. coelicolor* by AdpA, an AraC/XylS family transcription factor (Kim *et al.*, 2005; Kim *et al.*, 2008). Our microarray experiments (Huang *et al.*, 2005) and confirmatory RT-PCR experiments (Fig. 1B) had shown that *ramR*, another sporulation-associated gene (Ma and Kendall, 1994) known to be transcriptionally controlled by AdpA (Nguyen *et al.*, 2003), is also upregulated in *absB* mutant bacteria. Moreover, as we had observed for *sti1* transcripts, *ramR* mRNA also was not targeted by the ribonucleolytic action of purified AbsB/RNase III protein (Fig. S2C).

AdpA was discovered initially because of its effects on antibiotic biosynthesis and sporulation in *S. griseus* (Ohnishi *et al.*, 1999) and subsequently found to similarly affect these processes in *S. coelicolor* (Nguyen *et al.*, 2003; Takano *et al.*, 2003, Park *et al.*, 2009). The *S. coelicolor adpA* gene has also been called *bldH*, and production of the AdpA protein is controlled by *bldA* (Merrick, 1976; Lawlor *et al.*, 1987) – a sporulation-regulating gene encoding a tRNA that enables the translation of AdpA and other genes containing TTA codons, which are rare in streptomycetes (Takano *et al.*, 2003). Interestingly, the TTA condons of the *adpA* genes of all sequenced *Streptomyces* species are located at precisely the same position in the protein coding sequence, suggesting that their remarkably conserved location may have biological relevance.

The above observations and considerations raised the possibility that upregulation of sporulation-associated genes in *absB* mutant bacteria may be mediated by AdpA. Consistent with this notion, review of our microarray data (Huang *et al.*, 2005) and the *S. coelicolor* genome sequence (Bentley *et al.*, 2002) indicated that 20 *S. coelicolor* genes whose expression was increased or decreased more than sixfold by mutation of *absB* contain, 5′ to the ORF, a sequence showing 100% correspondence to the AdpA-binding motif (5′-TGGCSNGWY-3′ (S: G or C; W: A or T; Y: T or C) that has been found in the promoter regions of multiple *S. griseus* AdpA target genes (Yamazaki *et al.*, 2004). These genes and the positions of the AdpA-binding motifs relative to the start codon of the ORF are shown in Table S1.

**Fig. 2:**
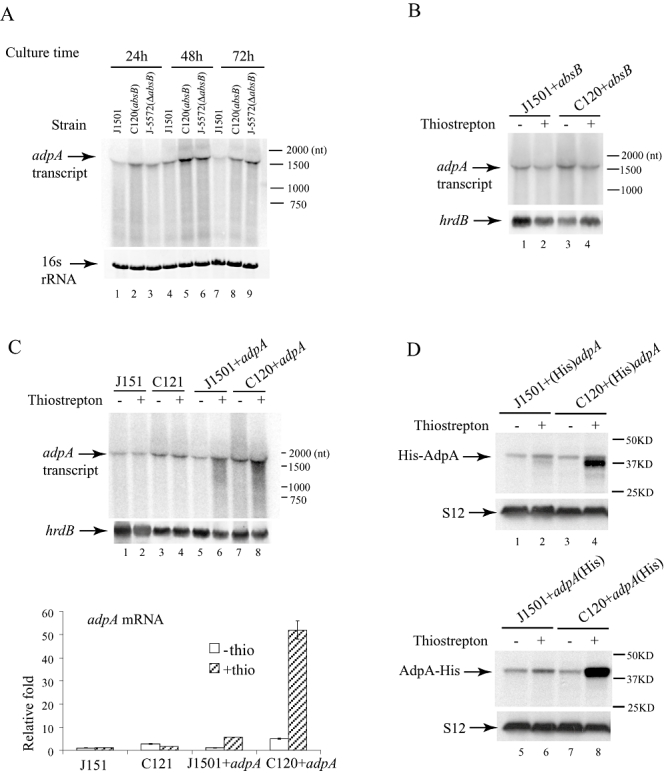
A. Northern blot analysis of *adpA* mRNA abundance in J1501 (morphologically wild type), C120 (*absB* point mutant) and J-5572 (Δ*absB*) strains of *S. coelicolor*. Mycelia were collected after 24, 48 or 72 h of incubation. The band corresponding in length to full-length *adpA* transcript is indicated by an arrow. 16srRNA transcripts indicated by lower arrow were used as internal loading controls. B. Northern blotting of *adpA* mRNA abundance in strains expressing *absB* adventitiously (J151: empty vector control in J1501; C121: empty vector control in C120; J1501 + *absB*: overexpression of *absB* in J1501; C120 + *absB*: overexpression of *absB* in C120) after growth on R5-aparamycin plates containing (+) or lacking thiostrepton (−) for 48 h. *hrdB* transcripts were the internal control. C. Quantitative RT-PCR analysis and Northern blotting of *adpA* mRNA abundance in strains overexpressing *adpA* (J151: empty vector control in J1501; C121: empty vector control in C120; J1501 + *adpA*: overexpression of *adpA* in J1501; C120 + *adpA*: overexpression of *adpA* in C120). *hrdB* transcripts were the internal control. D. Western blotting of 5′ and 3′ His-tagged AdpA protein [J1501 + (His)*adpA*: overexpression of 5′ His-tagged *adpA* in J1501; C120 + (His)*adpA*: overexpression of 5′ His-tagged *adpA* in C120; J1501 + *adpA*(His): overexpression of 3′ His-tagged *adpA* in J1501; C120 + *adpA*(His): overexpression of 3′ His-tagged *adpA* in C120]. Strains expressing *adpA* under the control of *tipA* promoter. Anti-His antibody was used to detect His-tagged AdpA protein abundance, and S12 protein abundance was used as the internal control.

*AdpA* promoter activity has been shown by earlier studies to be developmentally controlled, peaking just prior to the formation of aerial hyphae (Nguyen *et al.*, 2003). Our Northern blot analysis confirmed this finding, and also indicated that the steady state level of full-length *adpA* transcripts was increased in both the C120 *absB* point mutation strain and in *absB* null mutation strain J-5572 at all times tested (Fig. 2A). Moreover, expression of the *absB* ORF in C120 under control of the thiostrepton-inducible *tipA* promoter (C120 + *absB*) reversed the *adpA* transcript elevation otherwise seen in the *absB* mutant C120 (Fig. 2B, lanes 4 vs. 3). We also observed that the abundance of adventitiously overexpressed *adpA* mRNA (Fig. 2C) and protein (Fig. 2D) following induction of the *adpA* gene under control of the *tipA* promoter was greater in C120 *absB* mutant bacteria than in *absB*^+^ cells (Fig. 2C, lane 8 vs. lane 6; Fig. 2D, lane 4 vs. lane 2, lane 8 vs. lane 6).

**Fig. 3:**
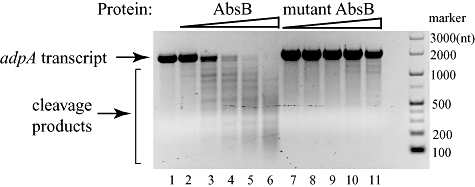
AbsB cleavage of *adpA* transcripts. 2 µg of *in vitro* transcribed *adpA* transcript was incubated with 6.25, 12.5, 25, 50, 100 ng of purified His-tagged AbsB protein (lanes 2–6) and the same amounts of mutant AbsB protein (lanes 7–11) that had been similarly purified as described. The arrows indicate the positions of uncleaved RNA and the cleavage products.

The notion that the AbsB/RNase III protein targets *adpA* transcripts ribonucleolytically was confirmed directly by demonstrating the ability of purified AbsB/RNase III protein to cleave a 1600 nt segment of *adpA* mRNA synthesized *in vitro* by bacteriophage T7 RNA polymerase on a template consisting of a PCR-amplified *adpA*-encoding segment of *S. coelicolor* genomic DNA (see *Experimental procedures*). As shown in Fig. 3, treatment of *adpA* transcripts with increasing amounts of wild type AbsB/RNase III protein resulted in decreasing intensity of the 1600 nt band corresponding to *adpA* transcript and yielded multiple degradation products (lanes 2–6) – whereas treatment of this transcript with ribonucleolytically inactive mis-sense mutant AbsB protein did not detectably affect the transcript's abundance or generate cleavage products (Fig. 3, lanes 7–11).

### Global effects of AbsB cleavage of *adpA* mRNA on *S. coelicolor* gene expression

**Fig. 4:**
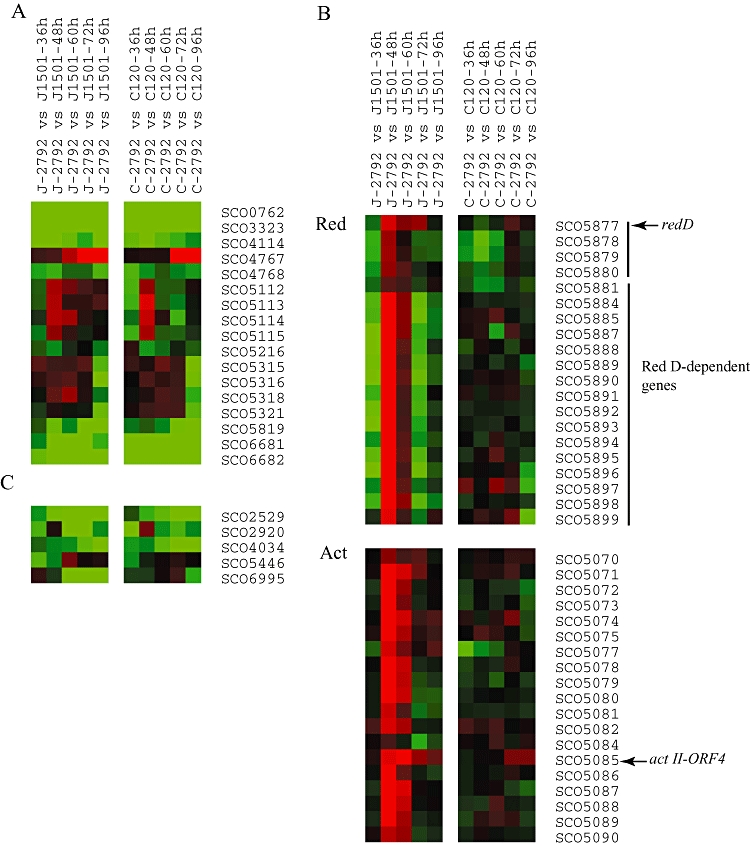
Microarray analysis of global transcriptional profile of J-2792 (Δ*adpA*) compared with J1501 (morphologically wild type) (left panel), and C-2792 (*absB*, Δ*adpA*) compared with C120 (*absB* mutant) (right panel). RNA samples from these four strains were isolated at the indicated time points (shown above the figure) during parallel growth on R5 media. Cy5-dCTP (red)-labelled cDNA corresponding to transcripts isolated from J-2792 (Δ*adpA*) were mixed with corresponding Cy3-dCTP (green)-labelled cDNA derived from J1501 (control strain transcripts obtained at the same time points); Cy5-dCTP labelled cDNA samples derived from the C-2792 (*absB*, Δ*adpA*) were hybridized with the same time point Cy3-dCTP labelled cDNA sample of C120 (*absB* mutant). Differences in transcript abundance for each gene (SCO designates are shown) are displayed by means of a colour scale, in which colour saturation represents the magnitude of the difference of RNA abundance between the detected strains (J-2792 or C-2792) and control strains (J1501 or C120) at the same indicated time point. The brighter red shades represent higher transcript abundance and brighter green shades represent lower transcript abundance in the detected stain comparing with the control strain (listed second among the strains pairs). Black indicates equal RNA abundance between the two strains, and grey represents the absence of data. Ratios of genes having multiple spots on the array were averaged. A. Selected sporulation-associated genes. B. Red and Act secondary metabolite pathway genes. C. Selected protease genes. Sporulation-associated and protease genes were selected on the basis of annotation information in StrepDB – The *Streptomyces* Annotation Server in the Sanger Institute.

Taken together, the results described argue strongly that accumulation of *adpA* transcripts in *absB* mutant bacteria (C120) underlies at least some of the previously observed phenotypic effects of *absB* mutations (Huang *et al.*, 2005). To learn more fully the extent to which cleavage of *adpA* mRNA by the AbsB/RNase III ribonuclease accounts for specific *absB*-mediated alterations in gene expression, we used DNA microarray analysis to investigate the effects of *adpA* mutations on gene expression in both parental (J1501) and *absB* mutant (C120) strains. Similar numbers of spores of J1501 (morphologically wild type), J-2792 (Δ*adpA*), C120 (*absB* point mutant) and C-2792 (*absB* point mutant, Δ*adpA*) were spread onto R5 solid medium. The total mass of mycelia was collected from each strain and weighed as an indicator of cell growth and/or proliferation, which was similar in all four strains (Fig. S3). RNA was harvested at various times after germination of spores (36, 48, 60, 72 or 96 h), and Cy5-dCTP red fluorescence-labelled cDNA from the J-2792 (Δ*adpA*) and C-2792 (*absB*, Δ*adpA*) strains was separately hybridized on DNA microarrays with Cy3-dCTP green fluorescence-labelled cDNA from strains J1501 and C120. Results from these experiments provided gene expression signatures for mycelium obtained at different stages of the bacterial growth cycle in the presence and absence of AdpA function (Fig. 4).

Analysis of the data using previously described methods (Huang *et al.*, 2005) indicated that *adp*A inactivation prevented expression of multiple other sporulation-related genes in both *absB*^+^ (J-2792 vs. J1501) or *absB* mutant (C-2792 vs. C120) bacteria (Fig. 4A). As was observed for *sti1*, expression of the *ramR* gene cluster (SCO6681,SCO6682) (Keijser *et al.*, 2002), and also of *bldN* (SCO3323) (Bibb *et al.*, 2000), *bldM* (SCO4768) (Molle and Buttner, 2000), SCO4114 (a sporulation-associated protein) (Li *et al.*, 2006) and *whiH* (SCO5819, a sporulation-related transcription factor) (Ryding *et al.*, 1998) were dramatically decreased in *adpA* mutant bacteria (J-2792) relative to J1501 levels at all time points tested (Fig. 4A). These results which are consistent with earlier studies showing that adventitious overexperession of *adpA* activates sporulation (Nguyen *et al.*, 2003), also show that AdpA is *necessary* for normal sporulation in *S. coelicolor*. Expression of the spore pigment-related *whiE* cluster (SCO5318-SCO5321) (Davis and Chater, 1990) was also reduced in *adpA* null mutant bacteria relative to the parental strain, although only at the 96 h time point.

**Fig. 5:**
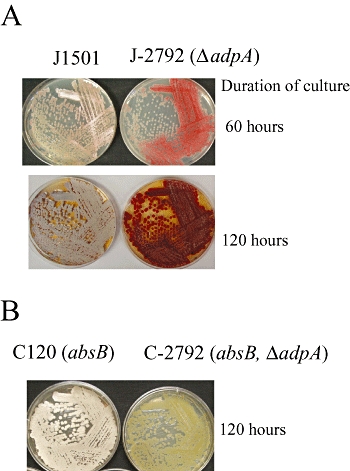
A. Phenotype of J-2792 (Δ*adpA*) compared with J1501 (morphologically wild type). B. Phenotype of C-2792 (*absB*, Δ*adpA*) compared with C120 (*absB* mutant). Cultures were grown on R5 medium for the duration shown on the right.

Inactivation of *adpA* in strain J-2792, a J1501 derivative, resulted in premature upregulation of multiple antibiotic biosynthesis genes (Fig. 4B), as has been reported previously for this lineage (Nguyen *et al.*, 2003). Whereas expression of Red and Act pathway-related genes normally is not activated until 60–72 h after the plating of J1501 spores onto R5 solid medium, and reaches a peak at 72–96 h (Huang *et al.*, 2005), in J-2792 (Δ*adpA*) expression of the Red and Act biosynthetic gene clusters was prematurely increased, as indicated by comparison of mRNA abundance relative to the morphologically wild type parental strain at the 48 h time point. Consistent with this finding, *adpA* inactivation resulted in premature production of pigmented antibiotics (Fig. 5A, upper panel). The premature turn on of the Red and Act genes in *adpA* null mutant bacteria suggests that the absence of expression of these genes early in the *S. coelicolor* life cycle is attributable in part to the action(s) of AdpA. The stimulatory effects of *adpA* mutation on antibiotic production in the J1501 lineage (Nguyen *et al.* 2003 and our current results) contrast with the inhibition of antibiotic production reported to result from *adpA* mutation in derivatives of *S. coelicolor* M145 (Takano *et al.*, 2003).

In *absB* mutant bacteria (C120), *adpA* transcripts and protein accumulate (Figs 2 and 3) and antibiotic biosynthesis gene transcripts are sharply decreased in abundance (Huang *et al.*, 2005). However, notwithstanding accumulation of AdpA in these bacteria, and the demonstrated ability of AdpA accumulation to downregulate antibiotic biosynthesis in this lineage (Nguyen *et al.*, 2003), introduction of an *adpA* null mutation into a J1501-derived *absB* mutant did not restore antibiotic synthesis (strain C-2792 mutated both *adpA* and *absB*; Fig. 5B). We therefore conclude that a mechanism other than enhanced AdpA repression of antibiotic biosynthetic genes underlies the defective antibiotic synthesis observed in *absB* mutant bacteria.

### AbsB and AdpA participate in a feedback-control regulatory loop

**Fig. 6:**
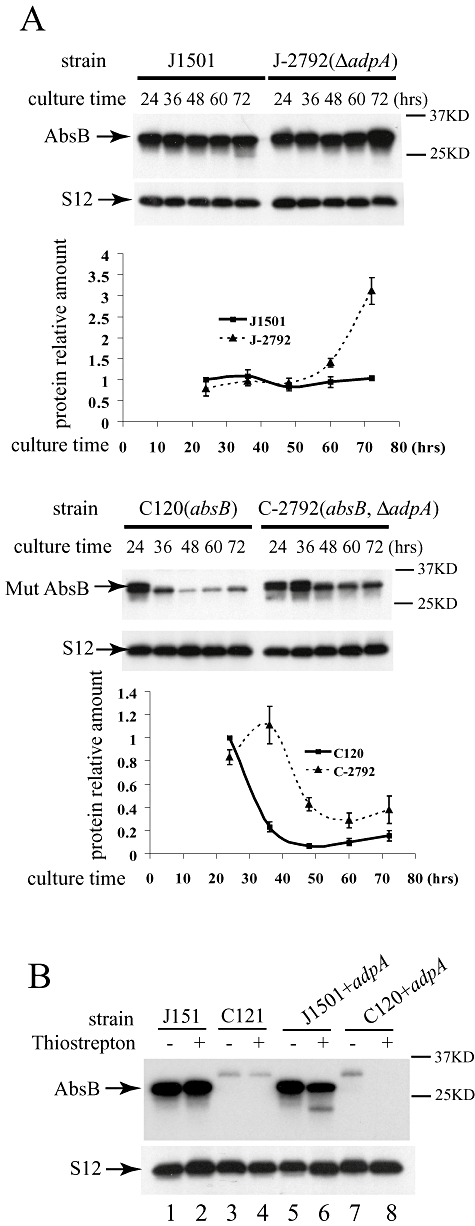
A. AbsB protein abundance in J1501 (morphologically wild type), J-2792 (Δ*adpA*), C120 (*absB* mutant), C-2792 (*absB*, Δ*adpA*) strains growing on R5 plates for 24–72 h as indicated. Western blotting was performed following the method in *Experimental procedures* using rabbit polyclonal antibody generated against purified AbsB protein. The protein abundances of S12 were used as loading control. The image shown is representative of three Western blotting experiments that were quantitatively analysed. B. Western blotting of AbsB protein abundance in J1501 and C120 strain expressing *adpA* adventitiously (J151: empty vector control in J1501; C121: empty vector control in C120 (*absB* mutant); J1501 + *adpA*: *adpA* overexpression in J1501; C120 + *adpA*: *adpA* overexpression in C120). Cultures were grown for 48 h on R5-aparamycin plates containing (+) or lacking (−) thiostrepton.

Previous studies from our lab have shown that the AbsB protein cleaves the transcript that encodes it and consequently that *absB* mRNA is elevated in *absB* mis-sense mutant bacteria (i.e. strain C120; Xu *et al.*, 2008). However, we observed paradoxically that despite elevation of mutant *absB* mRNA in strain C120, the abundance of the mutant AbsB protein was decreased at late times in the growth cycle (Mut AbsB, Fig. 6A, bottom panel), whereas the abundance of the wild type AbsB protein remained constant throughout the growth cycle (Fig. 6A, top panel). The decrease in mutant AbsB protein, which temporally correlates with the time of onset of enhanced AdpA synthesis (Nguyen *et al.* 2003 and Fig. 2A), was partially reversed by mutation of *adpA* (strain C-2792; *absB*, Δ*adpA*; Fig. 6A bottom panel). The notion that *adpA* expression can regulate the abundance of the AbsB protein was directly confirmed by experiments showing that adventitious overexpression of *adpA* under control of a thiostrepton-regulated *tipA* promoter incrementally decreased AbsB protein abundance (Fig. 6B).

### *adpA* mutation results in decreased expression of multiple protease genes

Western blot analysis using anti-AbsB antibody showed the occurrence of an immuno-reactive band of lower molecular weight than full-length AbsB protein in bacteria that adventitiously express AdpA (Fig. 6B, lane 6), consistent with the occurrence of AdpA-induced degradation of AbsB protein. In *S. griseus*, AdpA has in fact been shown to be an inducer of both intracellular and secreted proteases (Ohnishi *et al.*, 2005, Akanuma *et al.*, 2009), and our microarray analysis showed that in *S. coelicolor*, the abundance of RNAs encoding multiple proteases or putative proteases [SCO6995 (putative intracellular serine protease), SCO2529 (putative intracellular metalloprotease), SCO2920 (putative secreted protease) and SCO5446 (probable intracellular neutral zinc metalloprotease)] was dramatically decreased in *adpA* null mutant strains J-2792 (Δ*adpA*) and C-2792 (*absB*, Δ*adpA*) (Fig. 4C). Moreover, the SCO6995 and SCO5446 genes contain a sequence similar to the AdpA-binding motif in their putative promoter regions (Table S2).

## Discussion

**Fig. 7:**
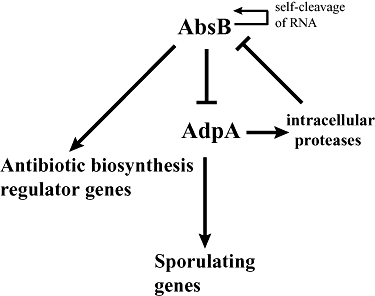
Proposed AbsB/AdpA autoregulatory loop. Arrows indicate positive control. Perpendicular lines indicate negative control.

Our results demonstrate that mRNA encoded by the multifunctional transcription factor AdpA is a target of the AbsB/RNase III protein of *S. coelicolor* and that the abundance of *adpA* mRNA and protein is modulated by AbsB cleavage of *adpA* transcripts. They further show that AdpA production is necessary for the stimulatory effects of *absB* mutations on sporulation-associated genes, and that AdpA in turn regulates AbsB actions by promoting degradation of the AbsB protein – possibly by initiating production of a still-unidentified protease. The AbsB/AdpA autoregulatory circuit (as depicted schematically in Fig. 7) thus constitutes a biologically complex pathway of transcriptional and translational regulation.

The discovery of AbsB resulted from the observation that *absB* mutant bacteria are defective in antibiotic synthesis (Adamidis and Champness, 1992; Aceti and Champness, 1998; Price *et al.*, 1999). Mutation of *absB* was also found to dramatically increase the abundance of certain sporulation-associated transcripts (Huang *et al.*, 2005). Although the phenotypic effect of *absB* mutation on sporulation is less dramatic than the effect on antibiotic biosynthesis, we observed that the *absB* mutant strain C120 generates thicker and greyer spores on rich R5 medium, and a larger fraction of these spores are biologically functional (Fig. S1). Our finding that introduction of an *adpA* mutation into *absB* mutant bacteria does not reverse the defect in antibiotic production (Fig. 5B), indicates that defective cleavage of *adpA* transcripts in *absB* mutant bacteria is not sufficient to account for the absence of antibiotic biosynthesis in these bacteria. This result, which contrasts with the effects of *adpA* mutation on the *absB*-dependent enhanced sporulation phenotype, suggests that separate mechanisms underlie the effects of AbsB on antibiotic synthesis and sporulation.

Consistent with the observed phenotypic effects of *adpA* null mutations, *adpA* function was required for the increase of sporulation-related transcripts observed in the *absB* mutant strain during microarray analysis (Fig. 4A). However, while *adpA* mutation leads to premature antibiotic synthesis, this event did not occur in the absence of the AbsB/RNase III gene product (Fig. 4B), suggesting that the AdpA regulation of antibiotic biosynthesis that we and others (Nguyen *et al.*, 2003) have observed in the J1501 lineage is dependent on AbsB. Together, these data lead us to suggest that the ability of AdpA excess to decrease antibiotic production may occur by AdpA-mediated downregulation of AbsB protein abundance (Fig. 6) occurring through the AbsB/AdpA regulatory loop described here (Fig. 7).

*OrnA* (SCO2793), the gene immediately 3′ to *adpA* encodes an orthologue of *Escherichia coli* oligonucleotidase, and in both *S. griseus* and *S. coelicolor* this gene has been reported to affect morphological differentiation and antibiotic generation (Ohnishi *et al.*, 2000; Sello and Buttner, 2008). In *S. coelicolor* strain M145, *adpA* mutation has been reported to have a polar effect on expression of *ornA* (Takano *et al.*, 2003), even though the two genes are separated by a 341 bp intergenic region (Bentley *et al.*, 2002). Notwithstanding this reported polarity, recent nuclease S1 analysis of transcriptional start sites within the *adpA/ornA* region has led to the conclusion that the two genes are not co-transcribed in this *Streptomyces* strain (Sello and Buttner, 2008). However, RT-PCR experiments carried out in another *S. coelicolor* strain J1501 during the investigations reported here, detected a transcript > 2000 nucleotides in length that includes sequences from both *adpA* and *ornA* (Fig. S4A). Additionally, the abundance of the bicistronic *adpA-ornA* transcript was greater in an *absB* derivative of J1501 strain at later times of culturing (from 60 h to 120 h) (Fig. S4A). Importantly, in *adpA* null mutant strains J-2792 (Δ*adpA*) and C-2792 (*absB*, Δ*adpA*), we observed that the abundance of *ornA*-containing transcripts in strains deleted for *adpA* is dramatically elevated during the life cycle (Fig. S4B), suggesting that read-through transcription from the *adpA* promoter may suppress transcription initiation from the intercistronic *ornA* promoter, and that the increase of *ornA* expression may contribute to the antibiotic-deficiency phenotype of the double null mutation strain C-2792 (*absB*, Δ*adpA*).

Although the work reported here is not intended to address in detail the mechanism underlying feedback control of AbsB by AdpA, the data we have presented argue that control occurs post-transcriptionally at the level of protein abundance. We hypothesize that AdpA may activate the transcription of genes encoding intracellular proteases that attack the AbsB protein, and that mutation of *absB*, which stabilizes *adpA* mRNA and leads to increased AdpA production (Fig. 2), increases intracellular protease gene transcription – and consequently accelerates AbsB protein degradation. Consistent with this model, transcripts of two putative protease genes (SCO5446, SCO6995) were elevated by adventitious expression of *adpA* under control of a thiostrepton-induced *tipA* promoter. We also observed that adventitious expression of these two genes inhibited antibiotic production (*data not shown*). However, we were unable to detect a direct effect of the proteases on AbsB protein degradation *in vivo*, indicating that their ability to reduce antibiotic biosynthesis is mediated by mechanism(s) other than targeting of the AbsB protein. Introduction of the SCO2529 and SCO2920 genes in the pIJ6902 vector into *E. coli* as a prelude to their transfer to and expression in *S. coelicolor* was not successful.

## Experimental procedures

### Strains and growth conditions

The strains used in this study are cited and referenced in Table 1. *S. coelicolor* strain J1501 and *absB* point mutation strain C120 were used in these studies. Strains expressing *absB* or *adpA* adventitiously from the inducible *tipA* promoter [J1501 + *absB*; C120 + *absB*; J1501 + *adpA*; C120 + *adpA*; J1501 + (His)*adpA*; J1501 + *adpA*(His); C120 + (His)*adpA*; C120 + *adpA*(His)] were constructed by introducing the apramycin resistance plasmid pIJ6902 containing the intact *absB*/*adpA* open reading frame (NdeI/BamHI) driven by the thiostrepton inducible *tipA* promoter into J1501 and C120 by conjugation (Huang *et al.*, 2005). *AbsB* and *adpA* null mutation strains were constructed by the insertional inactivation via double crossing over method as follows: the mutant allele was created by introducing an apramycin resistance gene cassette into a plasmid containing a thiostrepton resistance gene as a replacement for a DNA fragment carrying part of the target gene. The construct was transferred by conjugation into strains J1501 and C120, and apramycin resistant, thiostrepton sensitive clones were selected. The null mutations were confirmed by genomic PCR, real-time PCR and Southern blotting. The primers used for the construction of the null mutation strains and overexpression strains are listed in the Table S3.

**Table 1:** Strains used in this study.

Strain	Relevant Characteristic(s)	References
*S. coelicolor* A3(2)		
J1501	*hisA1 uraA1 strA1* SCP1– SCP2– Pgl–	Kieser *et al.* (2000)
C120	J1501 *absB120*	Price *et al.* (1999)
J-5572	J1501 Δ*absB*	This study
J-2792	J1501 Δ*adpA*	This study
C-2792	C120 Δ*adpA*	This study
J151	J1501 *tipAp::*	Huang *et al.* (2005)
C121	C120 *tipAp::*	Huang *et al.* (2005)
J1501 + *absB*	J1501 *tipAp::absB*	This study
C1501 + *absB*	C120 *tipAp::absB*	This study
J1501 + *adpA*	J1501 *tipAp::adpA*	This study
C120 + *adpA*	C120 *tipAp::adpA*	This study
J1501 + (His)*adpA*	J1501 *tipAp::*(His)*adpA*	THIS study
C120 + (His)*adpA*	C120 *tipAp::*(His)*adpA*	This study
J1501 + *adpA*(His)	J1501 *tipAp::adpA*(His)	This study
C120 + *adpA*(His)	C120 *tipAp::adpA*(His)	This study
*E. coli*		
DH5α		Invitrogen
ET12567/pUZ8002	*dam dcm*	Kieser *et al.* (2000)

For the growth on solid media, 150 µl of spore suspensions were inoculated at a density of OD_450_ = 0.06 onto cellophane membranes placed on R5 plates, and mycelia were collected from the cellophane surface at indicated time points. The growth of each strain was detected by measuring dry cell weight. 100 µg ml^−1^ apramycin and 50 µg ml^−1^ thiostrepton were added to the medium for the growth of thiostrepton-inducible overexpression strains.

### RNA extraction, Northern blotting, reverse transcription and quantitative PCR

Mycelia were collected from solid or liquid growth culture, immediately frozen by addition of liquid nitrogen, and ground into powder with mortar and pestle. Then 20 µg of total RNA isolated using the RNeasy® Plant Kit was treated with additional DNase I (10 Unit; QIAGEN) and electrophoresed on denaturing agarose gels (NorthernMax-Gly 10× Gel Prep/Running Buffer (Applied Biosystems). Following transfer to Zeta-probe (Bio-Rad) membranes, RNA was analysed by Northern blotting at 50°C in Ultrahyb hybridization buffer using randomly radiolabelled DNA containing the ORF sequence as probe (NorthernMax kit, Applied Biosystems). After one round of low-stringency wash (2XSSC, 0.1%SDS) at 25°C and three rounds of high-stringency wash (0.1XSSC, 0.1%SDS) at 50°C, membranes were exposed to phosphor-imaging (Typhoon, GE) for signal detection and quantification. For quantitative real-time RT-PCR, first strand cDNA synthesis was carried out using 2 µg of total RNA and SuperScript III (Invitrogen), following the manufacturer's instructions, and the Bio-Rad iCycler TM Real-Time PCR Detection System and iQTM SYBR Green Supermix Kit were used for the PCR amplification using 1–10 times diluted first strand reaction product as template. The PCR conditions were as follows: 94°C for 10 min, 40 cycles of 94°C for 30 s, 62°C for 30 s and 72°C for 30 s. The target cDNA was normalized internally to *hrdB* levels or 16S rRNA (samples were diluted 10^4^ times) (all the primers used for RT-PCR are listed in Table S3). For reverse transcription of *adpA-ornA* read-through transcript, Herculase II (Stratagene) was used (95°C for 3 min, 35 cycles of 94°C for 30 s, 67°C for 40 s and 72°C for 2 min).

### *In vitro* cleavage assay

The N-terminal hexahistidine tagged wild type and mutant AbsB proteins were produced in the protease-deficient *E. coli* strain BL21 by cloning the *absB* ORF in the expression plasmid pET28a, and purified by Ni-column chromatography as described before (Xu *et al.*, 2008). Protein concentration was determined by BCA assay (Pierce) and confirmed by electrophoresis of the purified samples on 12% SDS-polyacrylamide gel.

DNA corresponding to the full length *sti1, ramR* and *adpA* transcript was amplified from *S. coelicolor* genomic DNA using 5′ primers that included the sequence of the bacteriophage T7 promoter, the primer sequences for *sti1*, *ramR*, and *adpA in vitro* transcription are listed in Table S3. RNA substrates were synthesized using MEGAscript T7 kit (Applied Biosystems) and purified by RNeasy Mini Kit (QIAGEN). For *sti1, ramR* and *absB* locus transcripts (Xu *et al.*, 2008), 2 µg of RNA substrates were incubated with 25 ng of purified protein with RNase III buffer (Applied Biosystems) in 20 µl of reaction mixtures. Reaction mixtures were incubated at 37°C for 30 min then quenched by the addition of lysis buffer RLT from an RNeasy Mini Kit (QIAGEN). Cleavage products were purified using RNeasy Mini Kit (QIAGEN) and electrophoresed in a glyoxal-denatured 1.5% agarose gel using NorthernMax-Gly 10× Gel Prep/Running Buffer (Applied Biosystems). Visualization of the reaction was carried out by exposing gels to UV light. For *adpA* transcripts, 2 µg of RNA substrates were incubated with 6–100 ng of purified protein in 20 µl of reactions (Applied Biosystems RNase III buffer).

### Protein extraction and Western blotting

Purified AbsB protein was submitted to the company (Covance) to make rabbit polyclonal anti-AbsB antibody. For Western blotting, mycelia were collected from solid or liquid growth culture, and destructed by sonication in NP-40 lysis buffer [50 mM Tris-Cl (pH 8.0), 150 mM NaCl, 1%NP-40] with Complete Protease Inhibitor Cocktail (Roche). After quantification with BCA protein assay kit (Pierce), the protein was electrophoresed on a 12% SDS-polyacrylamide gel, electro-blotted onto a nitrocellulose membrane, and probed with anti-AbsB or monoclonal anti-His antibody (QIAGEN).

### Microarray analysis

10–15 µg of total RNA was denatured in the presence of 5–6 ng of 72%-GC-content hexamers (total 13 µl) at 75°C for 10–15 min and snap-cooled in ice water before addition of the remaining reaction components: 3 µl of Cy3–dCTP or Cy5–dCTP (Amersham Pharmacia Biotech) and 14 µl of a cocktail that included 6 µl of 5× Superscript II Buffer, 3 µl of DTT (0.1 M), 3 µl of dNTP (4 mM dATP, 4 mM dTTP, 10 mM dGTP and 0.5 mM dCTP) and 2 µl of Superscript II (Invitrogen). The reverse transcriptase reaction was carried out for 10 min at 25°C followed by 2 h at 42°C. After labelling, purification of cDNA, hybridization and washing were carried out as described previously (Huang *et al.*, 2001). Built-in functions of the Stanford Microarray Database (http://smd.stanford.edu/) were used to normalize and analyse microarray data.
