# Supplementary material for: Regulation of morphological differentiation in S. coelicolor by RNase III (AbsB) cleavage of mRNA encoding the AdpA transcription factor
Source: Mol Microbiol. 2010 Jan 3;75(3):781–91. doi: 10.1111/j.1365-2958.2009.07023.x (PMC2936110; doi:10.1111/j.1365-2958.2009.07023.x)
Supplement: Supplementary file 1 [file mmi0075-0781-SD1.pdf]

Figure S1

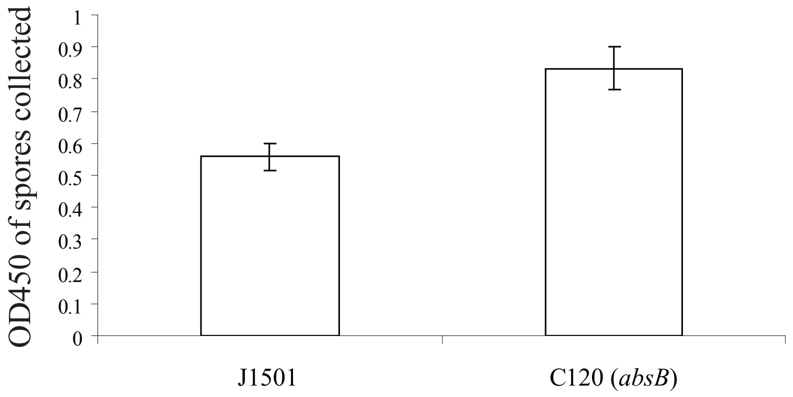
OD450 of spores collected
1
0.9
0.8
0.7
0.6
0.5
0.4
0.3
0.2
0.1
0
J1501
C120 (absB)

(A)

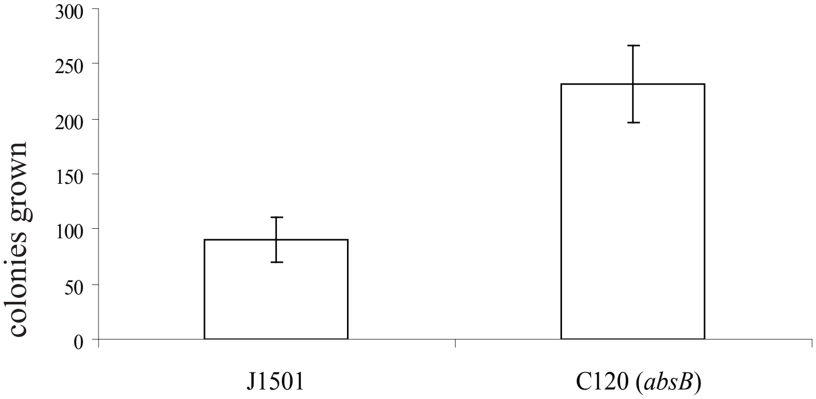
colonies grown
300
250
200
150
100
50
0
J1501
C120 (absB)

(B)

Figure S2

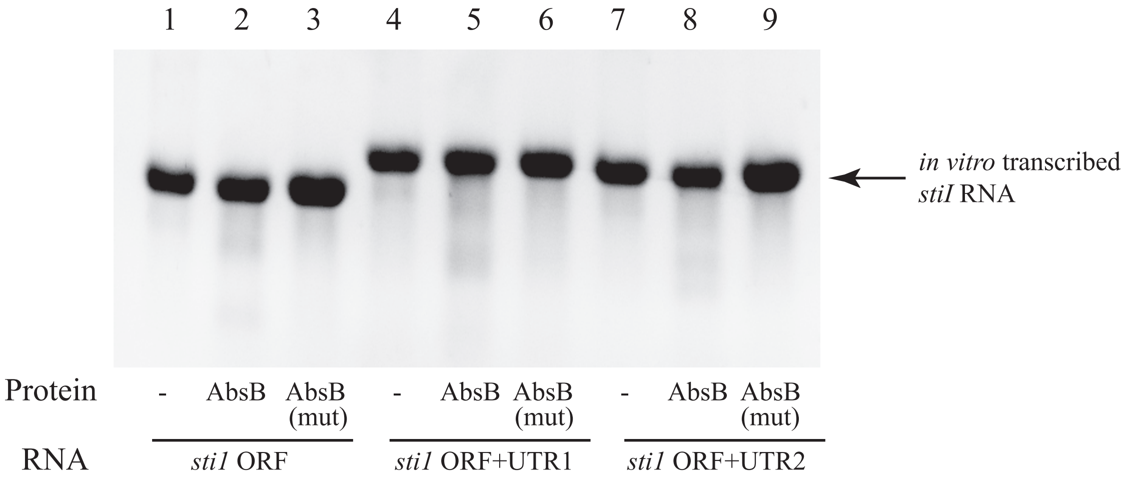

(A)

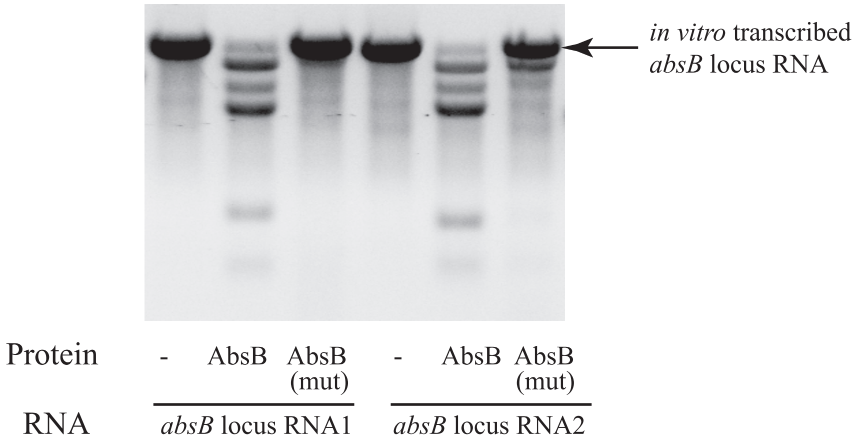

(B)

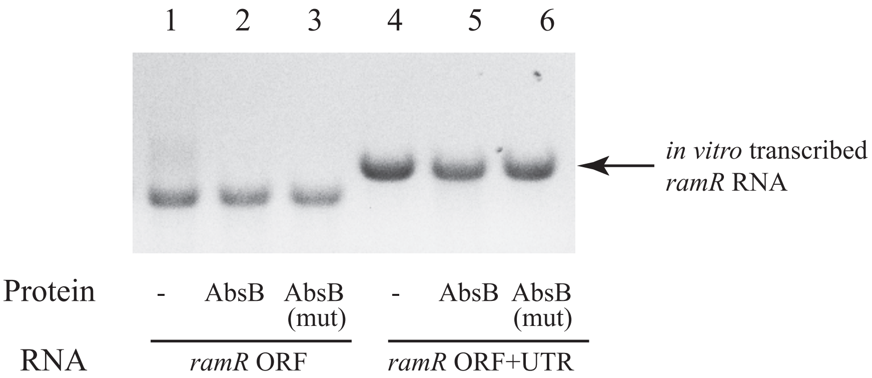

(C)

Figure S3

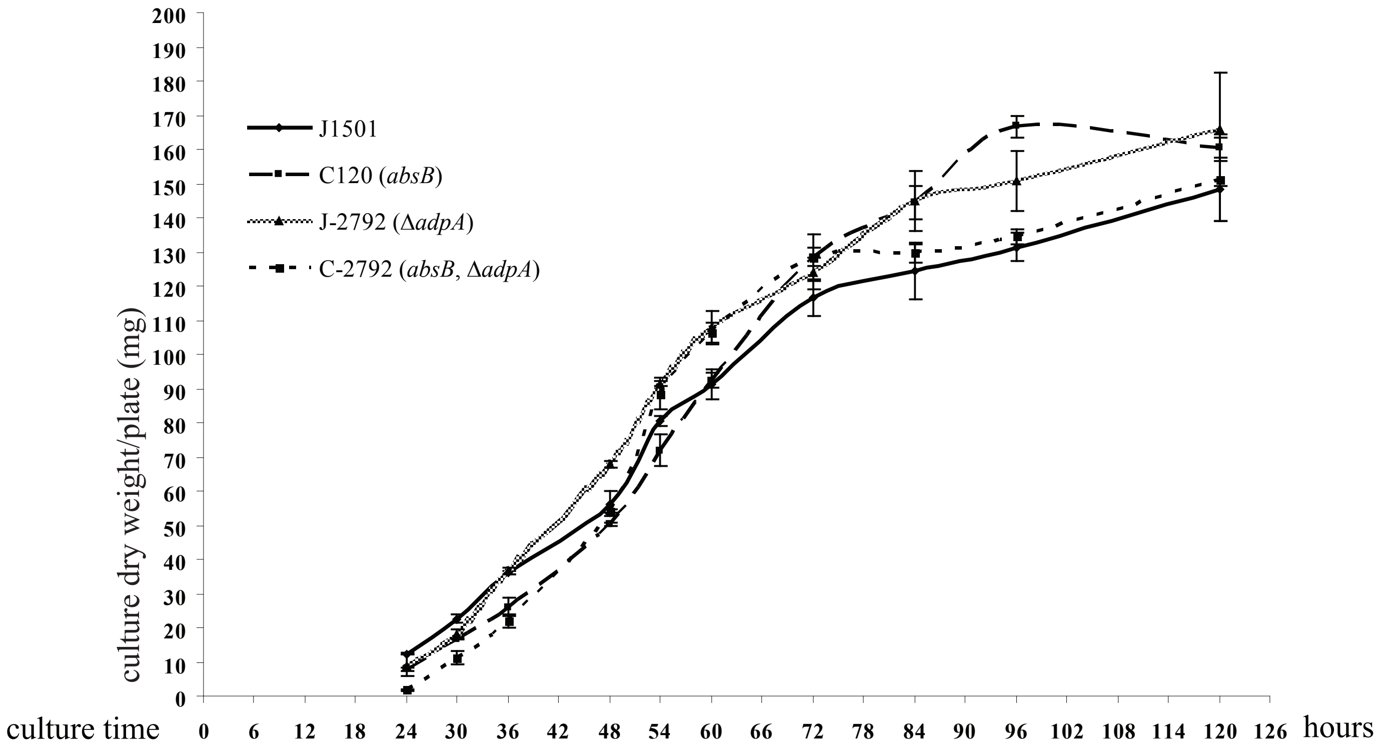
J1501
C120 (absB)
J-2792 (ΔadpA)
C-2792 (absB, ΔadpA)
culture dry weight/plate (mg)
200
190
180
170
160
150
140
130
120
110
100
90
80
70
60
50
40
30
20
10
0
culture time
0
6
12
18
24
30
36
42
48
54
60
66
72
78
84
90
96
102
108
114
120
126
hours

Figure S4

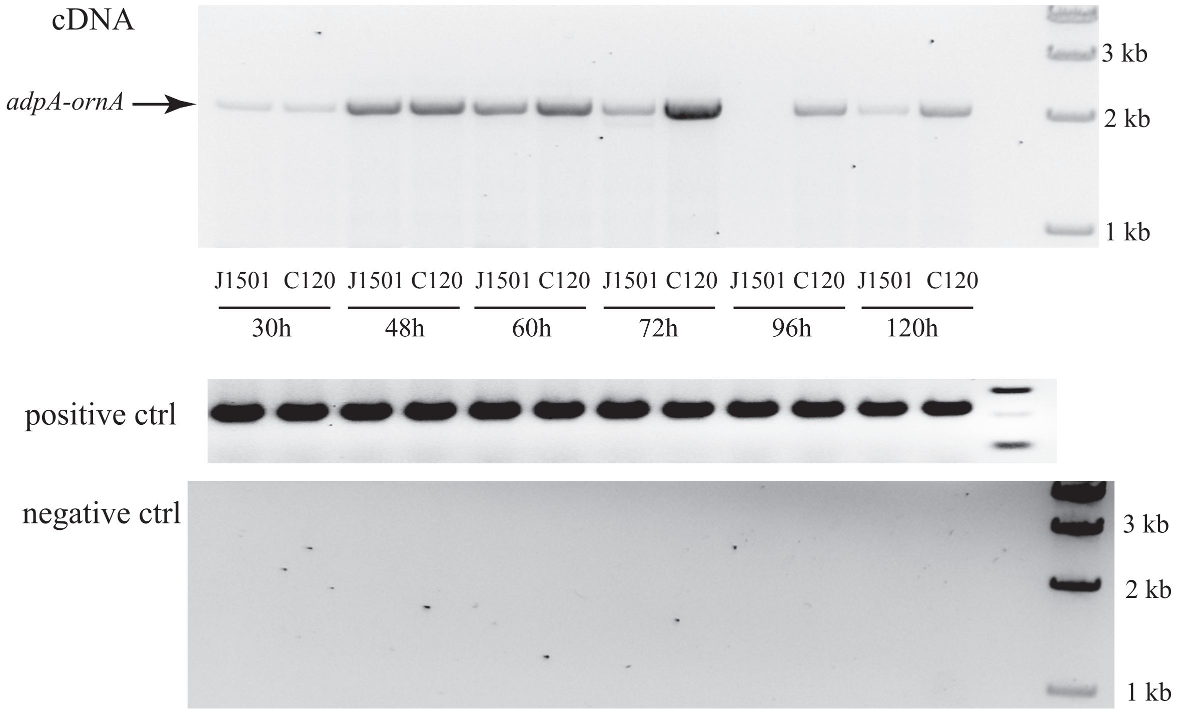
cDNA
adpA-ornA
3 kb
2 kb
1 kb
J1501 C120 J1501 C120 J1501 C120 J1501 C120 J1501 C120 J1501 C120
30h
48h
60h
72h
96h
120h
positive ctrl
negative ctrl
3 kb
2 kb
1 kb

(A)

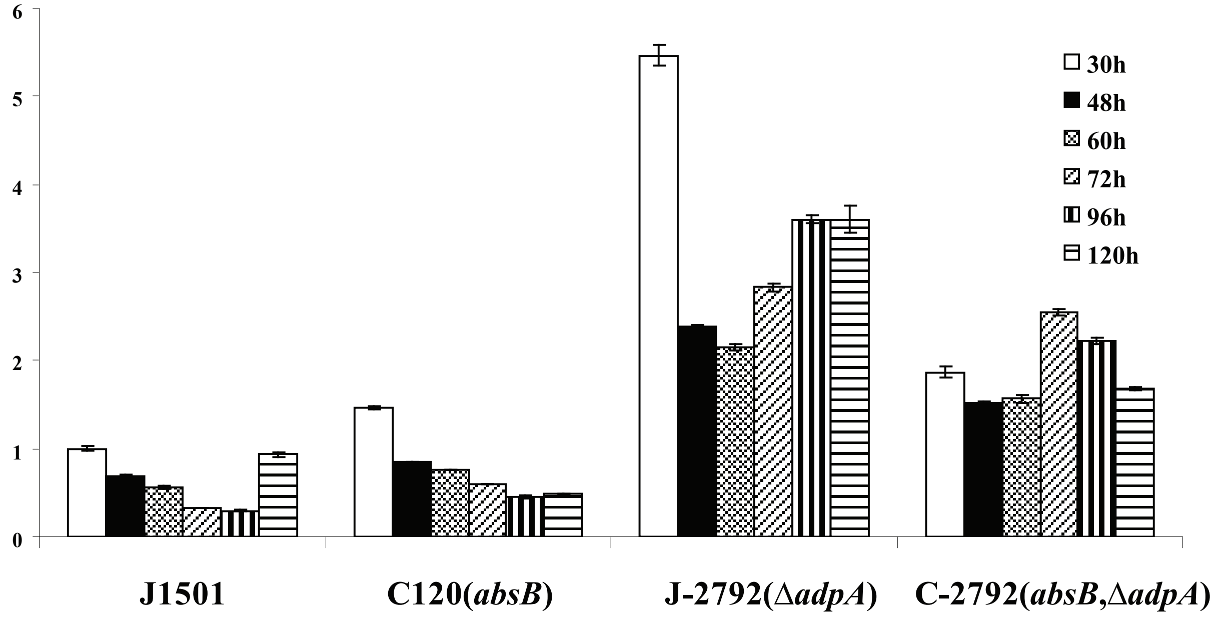
6
5
4
3
2
1
0
30h
48h
60h
72h
96h
120h
J1501
C120(absB)
J-2792(ΔadpA)
C-2792(absB,ΔadpA)

(B)

Supplementary Table 1

SCOID	Gene function	Motif	Location	Direction
SCO0072	hypothetical protein	tggccgaa	295	For
SCO0762	protease inhibitor precursor	tggcgtgat	233	Rev
SCO0762		tggccgaa	224	Rev
SCO0762		tggcgtgaa	150	Rev
SCO1401	conserved hypothetical protein	tggccgaa	225	Rev
SCO2239	putative secreted protein.	tggcccgat	133	Rev
SCO2633	superoxide dismutase [Fe-Zn] (EC 1.15.1.1)	tggcggat	8	Rev
SCO2714	putative secreted protein	tggcgtgat	71	For
SCO2913	conserved hypothetical protein	tggccggat	118	Rev
SCO2942	putative oxidoreductase (partial)	tggccgta	23	Rev
SCO3206	putative transmembrane efflux protein	tggcggta	114	Rev
SCO3731	cold-shock protein	tggcccgat	162	Rev
SCO4713	50S ribosomal protein L24	tggcggaa	75	Rev
SCO4840	putative secreted protein	tggccgta	60	For
SCO4947	nitrate reductase alpha chain NarG3	tggccgaa	225	For
SCO4948	nitrate reductase beta chain NarH3	tggccgaa	225	For
SCO5082	putative transcriptional regulatory protein	tggcccgtt	31	For
SCO5315	polyketide cyclase	tggccgtt	53	Rev
SCO5553	3-isopropylmalate dehydratase large subunit	tggcgcgaa	147	Rev
SCO6281	putative FAD-binding protein	tggccgaa	170	Rev
SCO6286	putative regulatory protein	tggcggaa	118	Rev
SCO6682	hypothetical protein SC5A7.32	tggcggaa	77	Rev

Supplementary Table 2

SCOID	Motif	Location	Direction	SCOID	Motif	Location	Direction	SCOID	Motif	Location	Direction	SCOID	Motif	Location	Direction
SCO0018	aacggcca	152	Rev	SCO0974	tggcctgtt	57	For	SCO1907	ttcggcca	136	Rev	SCO2788	ttccgcca	71	Rev
SCO0026	atcggcca	27	Rev	SCO0974	atccggcca	123	Rev	SCO1908	tggccgaa	87	For	SCO2791	tggccggtt	232	For
SCO0033	aaccggcca	55	Rev	SCO0975	tggccggat	201	For	SCO1913	tggcggat	63	For	SCO2791	tggcgtgaa	192	For
SCO0034	tggccggtt	219	For	SCO0975	aacaggcca	267	Rev	SCO1914	atccgcca	82	Rev	SCO2792	ttcacgcca	187	Rev
SCO0036	tggcccgta	28	For	SCO1009	tggcctgat	208	For	SCO1944	tggcgggta	145	For	SCO2792	aaccggcca	147	Rev
SCO0037	tggcccgta	28	For	SCO1031	tggccgta	82	For	SCO1945	tggcgggta	145	For	SCO2808	tacccgcca	89	Rev
SCO0038	tggcccgta	28	For	SCO1032	tggccgta	82	For	SCO1946	tggcgggta	145	For	SCO2809	tacccgcca	89	Rev
SCO0039	tacgggcca	225	Rev	SCO1033	tggccgta	82	For	SCO1947	tggcgggta	145	For	SCO2810	tacccgcca	89	Rev
SCO0040	tacgggcca	225	Rev	SCO1034	tacggcca	27	Rev	SCO1963	atcaggcca	127	Rev	SCO2813	tggccgta	43	For
SCO0041	tacgggcca	225	Rev	SCO1035	tacggcca	27	Rev	SCO2009	atccggcca	49	Rev	SCO2821	ttccgcca	75	Rev
SCO0042	tacgggcca	225	Rev	SCO1036	tacggcca	27	Rev	SCO2010	atccggcca	49	Rev	SCO2821	atcacgcca	51	Rev
SCO0059	aaccgcca	204	Rev	SCO1047	aaccgcca	173	Rev	SCO2011	atccggcca	49	Rev	SCO2844	aacggcca	53	Rev
SCO0059	aacggcca	89	Rev	SCO1047	aaccgcca	139	Rev	SCO2012	atccggcca	49	Rev	SCO2845	aacggcca	53	Rev
SCO0060	aaccgcca	204	Rev	SCO1047	aaccgcca	105	Rev	SCO2022	tggcggta	158	For	SCO2867	ttcgcgcca	164	Rev
SCO0060	aacggcca	89	Rev	SCO1047	aaccgcca	71	Rev	SCO2023	taccgcca	47	Rev	SCO2868	ttcgcgcca	164	Rev
SCO0062	tggccggat	259	For	SCO1084	tggccgaa	98	For	SCO2024	taccgcca	47	Rev	SCO2869	ttcgcgcca	164	Rev
SCO0062	atcgggcca	274	Rev	SCO1085	ttcggcca	174	Rev	SCO2025	tggccagaa	18	For	SCO2890	ttccgcca	192	Rev
SCO0063	tggccggat	259	For	SCO1103	atccggcca	8	Rev	SCO2026	tggccagaa	18	For	SCO2907	tggccggta	119	For
SCO0063	atcgggcca	274	Rev	SCO1131	tggccgaa	167	For	SCO2068	tggccggtt	91	For	SCO2912	atccggcca	117	Rev
SCO0064	tggccggat	259	For	SCO1132	tggccgaa	167	For	SCO2092	tggcgcgaa	248	For	SCO2913	atccggcca	117	Rev
SCO0064	atcgggcca	274	Rev	SCO1133	tggccgaa	167	For	SCO2093	ttcgcgcca	256	Rev	SCO2921	tggcggaa	78	For
SCO0065	tggcccgat	25	For	SCO1134	tggccgaa	167	For	SCO2093	atcccgcca	146	Rev	SCO2924	aacccgcca	8	Rev
SCO0065	atccggcca	40	Rev	SCO1135	ttcggcca	28	Rev	SCO2093	atccgcca	10	Rev	SCO2934	atcgcgcca	23	Rev
SCO0066	tggcccgat	25	For	SCO1143	tggcgggta	13	For	SCO2097	tacggcca	124	Rev	SCO2940	tacggcca	22	Rev
SCO0066	atccggcca	40	Rev	SCO1175	tggcggaa	245	For	SCO2102	aacggcca	24	Rev	SCO2941	tacggcca	22	Rev
SCO0067	tggcccgat	25	For	SCO1227	ttcggcca	113	Rev	SCO2104	atccgcca	141	Rev	SCO2942	tacggcca	22	Rev
SCO0067	atccggcca	40	Rev	SCO1228	tggccgaa	110	For	SCO2136	tggcggaa	125	For	SCO2966	ttccgcca	39	Rev
SCO0072	tggccgaa	294	For	SCO1231	tggccgat	30	For	SCO2138	tacccgcca	85	Rev	SCO3005	tggccgtt	67	For
SCO0073	ttcggcca	224	Rev	SCO1232	tggccgat	30	For	SCO2139	tggcgggta	80	For	SCO3006	aacggcca	221	Rev
SCO0083	atcacgcca	60	Rev	SCO1233	tggcgtgat	25	For	SCO2140	tggcgggta	80	For	SCO3029	tggcggaa	22	For
SCO0107	tggcgggat	80	For	SCO1234	tggcgtgat	25	For	SCO2178	tggccgaa	218	For	SCO3030	ttcacgcca	28	Rev
SCO0108	atcccgcca	126	Rev	SCO1235	tggcgtgat	25	For	SCO2179	ttcggcca	43	Rev	SCO3054	tggcggta	18	For
SCO0131	ttccgcca	216	Rev	SCO1236	tggcgtgat	25	For	SCO2187	aacggcca	22	Rev	SCO3055	taccgcca	100	Rev
SCO0132	tggcggaa	77	For	SCO1251	tggccgat	28	For	SCO2195	tggccagta	224	For	SCO3056	taccgcca	100	Rev
SCO0226	tggcggaa	143	For	SCO1252	tggccgat	28	For	SCO2195	tggccgat	214	For	SCO3057	tggccgta	34	For
SCO0233	aacgggcca	32	Rev	SCO1290	tggccggat	29	For	SCO2195	tactggcca	227	Rev	SCO3092	tggcggat	166	For
SCO0234	tggcccgtt	264	For	SCO1295	ttcgggcca	8	Rev	SCO2199	tggccgaa	92	For	SCO3096	tggccgat	126	For
SCO0235	tggcccgtt	264	For	SCO1334	tggccggaa	66	For	SCO2212	ttccggcca	71	Rev	SCO3102	atcggcca	14	Rev
SCO0245	ttcggcca	140	Rev	SCO1345	ttccggcca	253	Rev	SCO2238	atcgggcca	132	Rev	SCO3109	tacggcca	149	Rev
SCO0246	ttcggcca	140	Rev	SCO1346	ttccggcca	253	Rev	SCO2239	atcgggcca	132	Rev	SCO3146	tacggcca	128	Rev
SCO0247	tggcggaa	148	For	SCO1347	tggccggaa	20	For	SCO2240	atcgggcca	132	Rev	SCO3146	tacggcca	65	Rev
SCO0255	tggcggaa	116	For	SCO1375	atccggcca	238	Rev	SCO2241	tggcccgat	167	For	SCO3177	tggcggat	39	For
SCO0256	ttccgcca	204	Rev	SCO1388	tggccggat	88	For	SCO2249	tggcgtgaa	218	For	SCO3178	tggcggta	74	For
SCO0323	tggcctgat	36	For	SCO1400	ttcggcca	224	Rev	SCO2249	tggcccgta	82	For	SCO3179	tggcggta	74	For
SCO0373	tggcggat	127	For	SCO1401	ttcggcca	224	Rev	SCO2257	tacggcca	78	Rev	SCO3180	tggcggta	74	For
SCO0373	tggccgaa	76	For	SCO1402	ttcggcca	224	Rev	SCO2258	tacggcca	78	Rev	SCO3184	atccgcca	229	Rev
SCO0373	aaccgcca	12	Rev	SCO1403	tggccgaa	202	For	SCO2275	ttcggcca	23	Rev	SCO3185	tggcggat	92	For
SCO0374	atccgcca	269	Rev	SCO1403	atcggcca	118	Rev	SCO2276	ttcggcca	23	Rev	SCO3193	tggccgta	7	For
SCO0380	taccgcca	22	Rev	SCO1404	atcacgcca	23	Rev	SCO2277	ttcggcca	23	Rev	SCO3206	taccgcca	113	Rev
SCO0402	tggcccgaa	149	For	SCO1405	atcacgcca	23	Rev	SCO2284	tggcggat	33	For	SCO3250	taccgcca	67	Rev
SCO0431	tggccagaa	253	For	SCO1414	tggcggtt	97	For	SCO2285	atccgcca	83	Rev	SCO3251	taccgcca	67	Rev
SCO0432	ttctggcca	51	Rev	SCO1415	aaccgcca	167	Rev	SCO2311	tggccgtt	175	For	SCO3252	taccgcca	67	Rev
SCO0445	tggcggtt	13	For	SCO1423	atcggcca	117	Rev	SCO2311	tggcgcgaa	147	For	SCO3256	ttcgcgcca	47	Rev
SCO0446	tggcggtt	13	For	SCO1428	atcggcca	86	Rev	SCO2312	tggccgtt	175	For	SCO3270	tggcggta	39	For
SCO0447	aaccgcca	122	Rev	SCO1431	tggcggat	136	For	SCO2312	tggcgcgaa	147	For	SCO3302	taccggcca	74	Rev
SCO0505	aacgcgcca	169	Rev	SCO1432	tggcggat	136	For	SCO2313	tggccgtt	175	For	SCO3323	tggccgaa	107	For
SCO0510	tggcggta	63	For	SCO1433	atccgcca	145	Rev	SCO2313	tggcgcgaa	147	For	SCO3350	aacgcgcca	45	Rev
SCO0511	taccgcca	154	Rev	SCO1461	atcggcca	84	Rev	SCO2328	atcgcgcca	8	Rev	SCO3350	ttcgcgcca	35	Rev
SCO0529	aaccgcca	24	Rev	SCO1463	aacccgcca	90	Rev	SCO2346	tggcgcgaa	112	For	SCO3365	ttcacgcca	100	Rev
SCO0536	tggcgcgaa	293	For	SCO1464	aacccgcca	90	Rev	SCO2350	tggcggaa	113	For	SCO3395	ttctggcca	121	Rev
SCO0537	ttcgcgcca	87	Rev	SCO1492	ttcaggcca	172	Rev	SCO2363	taccggcca	53	Rev	SCO3414	ttcgggcca	54	Rev
SCO0538	ttcgcgcca	87	Rev	SCO1513	tggccagaa	89	For	SCO2364	tggccggta	162	For	SCO3415	tggcccgaa	190	For
SCO0539	ttcgcgcca	87	Rev	SCO1542	atcggcca	52	Rev	SCO2370	tacggcca	23	Rev	SCO3453	ttccgcca	151	Rev
SCO0540	ttcgcgcca	87	Rev	SCO1618	tggcgggaa	205	For	SCO2394	tggcgcgta	123	For	SCO3454	ttccgcca	151	Rev
SCO0550	ttccgcca	37	Rev	SCO1618	tggcgagaa	130	For	SCO2423	ttcggcca	285	Rev	SCO3455	ttccgcca	151	Rev
SCO0558	tggcggat	22	For	SCO1618	ttcggcca	267	Rev	SCO2446	tggcggaa	121	For	SCO3456	ttccgcca	151	Rev
SCO0605	ttccggcca	8	Rev	SCO1619	tggcgggaa	205	For	SCO2446	aacccgcca	114	Rev	SCO3457	tggcggaa	19	For
SCO0612	atcggcca	22	Rev	SCO1619	tggcgagaa	130	For	SCO2447	tggcgggtt	72	For	SCO3472	taccgcca	285	Rev
SCO0613	atcggcca	22	Rev	SCO1619	ttcggcca	267	Rev	SCO2447	ttccgcca	64	Rev	SCO3476	tggccgaa	292	For
SCO0618	tggccggaa	48	For	SCO1633	aacccgcca	267	Rev	SCO2448	tggcgggtt	72	For	SCO3476	aaccgcca	218	Rev
SCO0619	tggccggaa	48	For	SCO1648	atccggcca	209	Rev	SCO2448	ttccgcca	64	Rev	SCO3477	tggccgaa	292	For
SCO0638	ttcgcgcca	47	Rev	SCO1649	tggccggat	44	For	SCO2457	ttccggcca	67	Rev	SCO3477	aaccgcca	218	Rev
SCO0696	tggcgagat	111	For	SCO1655	atcggcca	79	Rev	SCO2458	ttccggcca	67	Rev	SCO3478	tggccgaa	292	For
SCO0697	atctcgcca	151	Rev	SCO1657	ttcggcca	281	Rev	SCO2459	tggccggaa	57	For	SCO3478	aaccgcca	218	Rev
SCO0754	ttccgcca	54	Rev	SCO1658	tggccgaa	129	For	SCO2461	tactcgcca	24	Rev	SCO3508	tggccgaa	36	For
SCO0755	ttccgcca	54	Rev	SCO1663	taccggcca	60	Rev	SCO2468	tggcctgat	86	For	SCO3521	tggcgggta	153	For
SCO0756	ttccgcca	54	Rev	SCO1669	tactggcca	100	Rev	SCO2469	tggcctgat	86	For	SCO3522	tggcgggta	153	For
SCO0757	ttccgcca	54	Rev	SCO1670	tggccagta	21	For	SCO2495	tggcgagaa	149	For	SCO3523	tacccgcca	166	Rev
SCO0762	atcacgcca	232	Rev	SCO1671	tggccagta	21	For	SCO2527	taccggcca	121	Rev	SCO3529	tggcggat	183	For
SCO0762	ttcggcca	223	Rev	SCO1676	tggccgat	108	For	SCO2527	atcacgcca	45	Rev	SCO3537	tggccggtt	195	For
SCO0762	ttcacgcca	149	Rev	SCO1676	ttccgcca	187	Rev	SCO2546	tggcggat	47	For	SCO3538	tggccggtt	195	For
SCO0763	tggcgtgaa	218	For	SCO1677	tggcggaa	148	For	SCO2547	tggcggat	47	For	SCO3539	aaccggcca	280	Rev
SCO0763	tggccgaa	143	For	SCO1677	atcggcca	227	Rev	SCO2548	tggcggat	47	For	SCO3543	tggccgtt	141	For
SCO0763	tggcgtgat	135	For	SCO1696	tggccggta	62	For	SCO2550	tggccgta	50	For	SCO3554	tggccggat	158	For
SCO0773	tggccgaa	83	For	SCO1704	atccgcca	43	Rev	SCO2551	tacggcca	62	Rev	SCO3555	tggccggat	158	For
SCO0773	atcgcgcca	177	Rev	SCO1704	ttctcgcca	24	Rev	SCO2571	tggccggaa	121	For	SCO3556	tggccggat	158	For
SCO0774	tggccgaa	83	For	SCO1708	ttcgcgcca	72	Rev	SCO2588	ttctcgcca	207	Rev	SCO3557	tggccggat	158	For
SCO0774	atcgcgcca	177	Rev	SCO1709	ttcgcgcca	72	Rev	SCO2619	aaccggcca	165	Rev	SCO3579	ttcgggcca	132	Rev
SCO0775	tggcgcgat	140	For	SCO1710	ttcgcgcca	72	Rev	SCO2619	atccgcca	156	Rev	SCO3614	tggcgggaa	206	For
SCO0775	ttcggcca	233	Rev	SCO1731	tggcggtt	61	For	SCO2620	atcccgcca	186	Rev	SCO3615	tggcgggaa	206	For
SCO0876	tggcgggat	27	For	SCO1732	aaccgcca	97	Rev	SCO2633	atccgcca	7	Rev	SCO3616	ttcccgcca	79	Rev
SCO0877	atcccgcca	52	Rev	SCO1776	tggccggaa	168	For	SCO2637	tggcggaa	164	For	SCO3629	tggcggaa	64	For
SCO0878	atcccgcca	52	Rev	SCO1792	tggccggtt	86	For	SCO2641	atcacgcca	8	Rev	SCO3630	ttccgcca	128	Rev
SCO0879	atcccgcca	52	Rev	SCO1793	aaccggcca	68	Rev	SCO2649	tggcggta	147	For	SCO3631	ttccgcca	128	Rev
SCO0880	tggcgtgat	219	For	SCO1805	atcccgcca	8	Rev	SCO2649	aactcgcca	97	Rev	SCO3661	tggccgaa	154	For
SCO0883	tggcgcgat	19	For	SCO1809	tggcctgtt	27	For	SCO2650	tggcggta	147	For	SCO3693	tggcggaa	114	For
SCO0898	tggcggat	28	For	SCO1839	atccgcca	290	Rev	SCO2650	aactcgcca	97	Rev	SCO3708	tggcggaa	45	For
SCO0899	atccgcca	190	Rev	SCO1842	tggcctgat	185	For	SCO2714	tggcgtgat	70	For	SCO3709	tggcggaa	45	For
SCO0942	tacggcca	7	Rev	SCO1857	ttccgcca	67	Rev	SCO2715	atcacgcca	82	Rev	SCO3710	ttccgcca	129	Rev
SCO0949	tggcgtgaa	86	For	SCO1858	ttccgcca	67	Rev	SCO2750	tacggcca	179	Rev	SCO3721	atcggcca	21	Rev
SCO0950	tggcgtgaa	86	For	SCO1859	ttccgcca	67	Rev	SCO2751	tacggcca	179	Rev	SCO3731	atcgggcca	161	Rev
SCO0951	tggcgtgaa	86	For	SCO1860	atccgcca	86	Rev	SCO2752	tacggcca	179	Rev	SCO3762	tacgcgcca	31	Rev
SCO0952	tggcgtgaa	86	For	SCO1878	atccggcca	52	Rev	SCO2753	tggccgta	58	For	SCO3774	atccggcca	133	Rev
SCO0953	ttcacgcca	155	Rev	SCO1891	aaccggcca	30	Rev	SCO2764	aacggcca	30	Rev	SCO3842	aaccgcca	7	Rev
SCO0966	ttccgcca	44	Rev	SCO1892	tggccggtt	119	For	SCO2765	tggccgtt	55	For	SCO3888	tggcggta	177	For

SCOID	Motif	Location	Direction
SCO3905	aacggcca	250	Rev
SCO3906	tggccgtt	32	For
SCO3917	tggccgaa	70	For
SCO3941	tggcccgat	114	For
SCO3942	atcgggcca	91	Rev
SCO3968	tggcggat	149	For
SCO3972	tggccggta	201	For
SCO3998	ttcggcca	41	Rev
SCO3999	ttccgcca	7	Rev
SCO4001	ttccgcca	89	Rev
SCO4002	tggcggaa	283	For
SCO4046	tggccgaa	31	For
SCO4047	tggccgaa	31	For
SCO4048	tggccgaa	31	For
SCO4049	ttcggcca	60	Rev
SCO4050	ttcggcca	60	Rev
SCO4051	ttcggcca	60	Rev
SCO4052	ttcggcca	60	Rev
SCO4070	ttcggcca	127	Rev
SCO4096	tggcgcgtt	82	For
SCO4097	ttcggcca	62	Rev
SCO4114	atcccgcca	114	Rev
SCO4131	aacccgcca	118	Rev
SCO4132	tggcgggtt	272	For
SCO4136	atccggcca	155	Rev
SCO4137	tggccggat	60	For
SCO4138	tggccggat	60	For
SCO4187	ttctcgcca	142	Rev
SCO4188	tggcgagaa	19	For
SCO4203	taccgcca	146	Rev
SCO4204	tggcggta	8	For
SCO4205	tggcggta	8	For
SCO4213	aacccgcca	131	Rev
SCO4284	tacgggcca	57	Rev
SCO4285	tacgggcca	57	Rev
SCO4286	tggcccgta	98	For
SCO4287	tggcccgta	98	For
SCO4289	aacccgcca	8	Rev
SCO4295	ttctcgcca	192	Rev
SCO4328	ttccgcca	51	Rev
SCO4329	tggccgta	196	For
SCO4330	tacggcca	144	Rev
SCO4344	ttccgcca	159	Rev
SCO4371	atcggcca	58	Rev
SCO4391	tggcggtt	113	For
SCO4392	aaccgcca	68	Rev
SCO4412	ttctggcca	83	Rev
SCO4413	tggcccgaa	46	For
SCO4413	atcgggcca	20	Rev
SCO4414	tggcccgat	72	For
SCO4414	ttcgggcca	46	Rev
SCO4415	tggcccgat	72	For
SCO4415	ttcgggcca	46	Rev
SCO4441	tggcgtgta	92	For
SCO4442	atcacgcca	63	Rev
SCO4500	atccgcca	21	Rev
SCO4501	atccgcca	21	Rev
SCO4502	tggcggat	148	For
SCO4562	aaccgcca	41	Rev
SCO4563	aaccgcca	41	Rev
SCO4564	aaccgcca	41	Rev
SCO4565	aaccgcca	41	Rev
SCO4566	aaccgcca	41	Rev
SCO4567	aaccgcca	41	Rev
SCO4568	aaccgcca	41	Rev
SCO4569	aaccgcca	41	Rev
SCO4570	aaccgcca	41	Rev
SCO4571	aaccgcca	41	Rev
SCO4572	aaccgcca	41	Rev
SCO4573	aaccgcca	41	Rev
SCO4574	aaccgcca	41	Rev
SCO4575	aaccgcca	41	Rev
SCO4579	tacggcca	46	Rev
SCO4589	tggccggat	110	For
SCO4596	tggccgaa	199	For
SCO4596	aaccggcca	213	Rev
SCO4617	atctcgcca	66	Rev
SCO4618	atctcgcca	66	Rev
SCO4628	tggcctgaa	89	For
SCO4629	tggcctgaa	89	For
SCO4631	tggcccgat	227	For
SCO4652	tggccgaa	191	For
SCO4712	ttccgcca	74	Rev
SCO4713	ttccgcca	74	Rev
SCO4714	ttccgcca	74	Rev
SCO4715	ttccgcca	74	Rev
SCO4803	atcggcca	7	Rev
SCO4812	tggccggaa	152	For
SCO4813	ttccggcca	150	Rev
SCO4814	ttccggcca	150	Rev
SCO4820	ttctcgcca	124	Rev
SCO4820	atcacgcca	115	Rev
SCO4821	ttctcgcca	124	Rev
SCO4821	atcacgcca	115	Rev
SCO4840	tggccgta	59	For
SCO4861	tggccgat	81	For
SCO4866	tggcggtt	162	For
SCO4867	tggcggtt	162	For
SCO4884	aaccggcca	123	Rev
SCO4946	taccgcca	98	Rev
SCO4947	tggccgaa	224	For
SCO4948	tggccgaa	224	For
SCO4949	tggccgaa	224	For
SCO4950	tggccgaa	224	For
SCO4960	tggcctgat	49	For

SCOID	Motif	Location	Direction
SCO4984	tggcgcgaa	115	For
SCO4985	ttcgcgcca	126	Rev
SCO5002	tggcggaa	10	For
SCO5057	tggcgggaa	70	For
SCO5058	ttcccgcca	66	Rev
SCO5082	tggcccgtt	30	For
SCO5083	aacgggcca	88	Rev
SCO5084	aacgggcca	88	Rev
SCO5104	ttcaggcca	211	Rev
SCO5105	atccggcca	187	Rev
SCO5109	ttccgcca	56	Rev
SCO5110	tggcggaa	123	For
SCO5128	tggccagta	79	For
SCO5167	tggccgat	82	For
SCO5167	atcgggcca	198	Rev
SCO5174	tggcgcgat	112	For
SCO5174	atcgcgcca	91	Rev
SCO5183	ttccgcca	262	Rev
SCO5184	ttccgcca	262	Rev
SCO5185	ttccgcca	262	Rev
SCO5239	taccgcca	23	Rev
SCO5240	tggcggta	90	For
SCO5247	tggccggaa	96	For
SCO5263	atctcgcca	193	Rev
SCO5281	ttcccgcca	150	Rev
SCO5286	ttccgcca	212	Rev
SCO5287	tggcggaa	43	For
SCO5303	tggcggtt	46	For
SCO5314	aacggcca	52	Rev
SCO5315	aacggcca	52	Rev
SCO5316	aacggcca	52	Rev
SCO5331	tggcggat	205	For
SCO5332	atccgcca	24	Rev
SCO5386	ttcggcca	110	Rev
SCO5388	atcggcca	25	Rev
SCO5389	tggccgat	178	For
SCO5427	tggccgaa	41	For
SCO5440	tggccggat	281	For
SCO5441	tggccggat	281	For
SCO5442	tggccggat	281	For
SCO5443	tggccggat	281	For
SCO5444	atccggcca	91	Rev
SCO5445	ttcggcca	134	Rev
SCO5446	ttcggcca	134	Rev
SCO5498	aacggcca	120	Rev
SCO5499	aacggcca	120	Rev
SCO5500	aacggcca	120	Rev
SCO5501	aacggcca	120	Rev
SCO5537	tggccgta	65	For
SCO5538	tggccgta	65	For
SCO5552	tggcgcgtt	59	For
SCO5553	aacgcgcca	146	Rev
SCO5554	aacgcgcca	146	Rev
SCO5555	ttcggcca	239	Rev
SCO5557	aaccgcca	54	Rev
SCO5557	aaccgcca	54	Rev
SCO5558	tggcggtt	96	For
SCO5559	tggcggtt	96	For
SCO5564	atcgggcca	115	Rev
SCO5565	tggcccgat	159	For
SCO5589	tggccggaa	296	For
SCO5589	atccggcca	239	Rev
SCO5589	atctcgcca	160	Rev
SCO5605	atccggcca	61	Rev
SCO5618	atcggcca	259	Rev
SCO5624	tacggcca	7	Rev
SCO5643	atcaggcca	9	Rev
SCO5644	tggcctgat	82	For
SCO5660	atccgcca	72	Rev
SCO5667	atcacgcca	101	Rev
SCO5668	atcacgcca	101	Rev
SCO5669	atcacgcca	101	Rev
SCO5670	atcacgcca	101	Rev
SCO5671	atcacgcca	101	Rev
SCO5723	ttccgcca	77	Rev
SCO5724	tggcggaa	233	For
SCO5725	tggcggaa	233	For
SCO5752	atcggcca	54	Rev
SCO5753	atcggcca	54	Rev
SCO5754	atcggcca	54	Rev
SCO5828	tacgcgcca	22	Rev
SCO5829	tacgcgcca	22	Rev
SCO5848	ttcgggcca	53	Rev
SCO5849	ttcgggcca	53	Rev
SCO5853	ttcggcca	148	Rev
SCO5854	tggccgaa	94	For
SCO5861	tggccggat	23	For
SCO5862	tggccgaa	99	For
SCO5863	tggccgaa	99	For
SCO5914	aaccggcca	144	Rev
SCO5915	tggccggtt	106	For
SCO5928	atcgggcca	83	Rev
SCO5933	tacggcca	112	Rev
SCO5934	tggccgta	122	For
SCO5935	tggccgta	122	For
SCO5970	ttcaggcca	139	Rev
SCO5999	tggcggta	102	For
SCO5999	atcggcca	63	Rev
SCO6003	tggcccgtt	43	For
SCO6015	atcacgcca	139	Rev
SCO6016	tggcgtgat	28	For
SCO6018	tggcgtgat	28	For
SCO6026	ttccggcca	179	Rev
SCO6027	ttccggcca	179	Rev
SCO6056	ttcccgcca	177	Rev

SCOID	Motif	Location	Direction
SCO6093	tggcgcgat	145	For
SCO6094	tggcgcgat	145	For
SCO6095	tggcgcgat	145	For
SCO6096	tggcgcgat	145	For
SCO6104	tggccgta	152	For
SCO6108	tggcggaa	243	For
SCO6109	tggcggaa	243	For
SCO6150	ttccgcca	42	Rev
SCO6151	ttccgcca	42	Rev
SCO6153	tggccgtt	68	For
SCO6153	ttcccgcca	61	Rev
SCO6162	tggcggat	23	For
SCO6163	tggcggat	23	For
SCO6193	tggcccgtt	100	For
SCO6234	aacggcca	245	Rev
SCO6281	ttcggcca	169	Rev
SCO6286	atccgcca	117	Rev
SCO6287	tggcggat	127	For
SCO6313	tggccgaa	130	For
SCO6314	tggccgaa	130	For
SCO6318	tggccagtt	160	For
SCO6319	aactggcca	259	Rev
SCO6337	ttccgcca	99	Rev
SCO6338	ttccgcca	99	Rev
SCO6339	tggcggaa	221	For
SCO6342	tggcggta	27	For
SCO6343	tggcggta	27	For
SCO6348	ttcccgcca	81	Rev
SCO6349	tggcgggaa	120	For
SCO6374	tggcgtgtt	23	For
SCO6374	tacggcca	166	Rev
SCO6377	atcggcca	25	Rev
SCO6382	aacggcca	153	Rev
SCO6383	tacggcca	239	Rev
SCO6385	tggcgtgta	90	For
SCO6386	tacacgcca	89	Rev
SCO6388	atcggcca	264	Rev
SCO6389	atcggcca	264	Rev
SCO6390	tggccgat	218	For
SCO6414	ttccggcca	89	Rev
SCO6415	ttccggcca	89	Rev
SCO6416	ttccggcca	89	Rev
SCO6461	atcccgcca	54	Rev
SCO6469	tggccgtt	285	For
SCO6470	tggccgtt	285	For
SCO6471	tggccgtt	285	For
SCO6472	tggccgtt	285	For
SCO6473	tggccgtt	285	For
SCO6481	ttcgggcca	207	Rev
SCO6482	tggcccgaa	37	For
SCO6486	ttcgggcca	8	Rev
SCO6487	ttcgggcca	8	Rev
SCO6588	ttccgcca	166	Rev
SCO6589	ttcgggcca	8	Rev
SCO6642	ttccggcca	50	Rev
SCO6643	tggccggaa	221	For
SCO6677	tggcggaa	78	For
SCO6678	tggcggaa	78	For
SCO6679	tggcggaa	78	For
SCO6680	tggcggaa	78	For
SCO6681	ttccgcca	76	Rev
SCO6682	ttccgcca	76	Rev
SCO6683	ttccgcca	76	Rev
SCO6684	ttccgcca	76	Rev
SCO6685	tggcccgaa	29	For
SCO6686	tggcgcgaa	145	For
SCO6687	tggcgcgaa	145	For
SCO6718	tggcgcgaa	80	For
SCO6735	tggcccgta	19	For
SCO6822	tggccggat	174	For
SCO6823	tggccggat	174	For
SCO6846	atctcgcca	46	Rev
SCO6910	atcacgcca	248	Rev
SCO6911	tggcgtgat	28	For
SCO6917	tggccgaa	57	For
SCO6994	tggccgat	27	For
SCO6995	atcggcca	124	Rev
SCO7000	tggccggaa	93	For
SCO7016	atcacgcca	264	Rev
SCO7017	atcacgcca	264	Rev
SCO7112	tggcggtt	209	For
SCO7113	aaccgcca	29	Rev
SCO7125	atccgcca	61	Rev
SCO7126	tggcggat	225	For
SCO7134	ttccggcca	189	Rev
SCO7171	tggccgat	183	For
SCO7171	tggccgat	91	For
SCO7172	atcggcca	235	Rev
SCO7172	atcggcca	143	Rev
SCO7173	tggccgat	121	For
SCO7187	tggccgta	126	For
SCO7188	tggccgta	108	For
SCO7191	tggccgaa	230	For
SCO7191	tggcgggaa	66	For
SCO7192	ttcccgcca	200	Rev
SCO7192	ttcggcca	35	Rev
SCO7195	tggcggta	201	For
SCO7211	tggcggtt	271	For
SCO7212	tggcggtt	271	For
SCO7258	tggccggaa	38	For
SCO7259	ttccggcca	112	Rev
SCO7272	tggccgaa	152	For
SCO7272	ttccggcca	188	Rev
SCO7273	tggccggaa	154	For
SCO7273	ttcggcca	189	Rev

SCOID	Motif	Location	Direction
SCO7319	tggccgaa	35	For
SCO7339	tactcgcca	49	Rev
SCO7340	tggcgagta	48	For
SCO7407	ttccggcca	29	Rev
SCO7408	ttccggcca	29	Rev
SCO7511	tggcgtgat	107	For
SCO7526	tggcgggaa	52	For
SCO7573	tggccgaa	139	For
SCO7574	ttcggcca	68	Rev
SCO7581	atccgcca	39	Rev
SCO7582	atccgcca	39	Rev
SCO7583	tggcggat	77	For
SCO7587	aacgggcca	13	Rev
SCO7588	atcggcca	144	Rev
SCO7631	taccgcca	119	Rev
SCO7632	tggcggta	131	For
SCO7657	atccggcca	138	Rev
SCO7658	atccggcca	138	Rev
SCO7659	ttcggcca	241	Rev
SCO7660	tggccgaa	92	For
SCO7661	tggccgaa	92	For
SCO7737	atccgcca	44	Rev
SCO7760	aactggcca	152	Rev
SCO7761	tggccagtt	51	For
SCO7791	tggcggat	50	For
SCO7791	tggcggat	50	For
SCO7829	aacggcca	153	Rev

Supplementary Table 3

T7-SCO0762-ORF-5' TAA TAC GAC TCA CTA TAG GGT TCG GTG CGG GAC TCG GCA GAC TCG C
T7-SCO0762-ORF-3' ACC GGG AGA ACG GCG GAC GGC CCC AC

T7-SCO0762-ORF+UTR-1-5' TAA TAC GAC TCA CTA TAG GGG GTT ACC GAA ACC CGT GGG GTC GAG
T7-SCO0762-ORF+UTR-1-3' GCC GAC CGT GCC CCT GAA CCG GGA G

T7-SCO0762-ORF+UTR-2-5' TAA TAC GAC TCA CTA TAG GGA TGA GTT TCG GTG CGG GAC TCG GCA
T7-SCO0762-ORF+UTR-2-3' GCC GAC CGT GCC CCT GAA CCG GGA G

SCO0762 real time-5' CCTCGGCCCTGGTGCTGAC
SCO0762 real time-3' TACTCCCGGGTGCACATCAC

SCO2792 real time -5’ AGG AAG CAC TCG ACG CAC TG
SCO2792 real time -3’ GCC GTC GTC CAC GAA GAG TT

SCO2792 cloning -5’+NdeI AAT TCA TAT GAG CCA CGA CTC CAC CGC CGC GCC G
SCO2792 cloning -3’+BamHI AAA GGA TCC ACC TCA CGG CGC GCT GCG CTG GCC

T7-SCO2792-5’ TAA TAC GAC TCA CTA TAG GGC GGC GCG GCG GGA CAC AGT AGA TT
T7-SCO2792-3’ CTG AAT TGC CCT GCC CGG CAT CGG

SCO2792 KN PCR1-5’ CGCGGGTTCGAAGAATTCGTGACC
SCO2792 KN PCR1-3’ AAAAAGCTTCGGTGGAGTCGTGGCTCATACTGC

SCO2792 KN PCR2-5’ AAAAAGCTTGCCGTCCCCAGGGCGACCGCCAGC
SCO2792 KN PCR2-3’ AAAAGATCTAGCTGAGCTACACCCACCAAGGCC

SCO2793 real time-5’ CACCGACTCCGAGCTGAACA
SCO2793 real time-3’: TCACGTGCTCCCGCACATAG

SCO5570 real time-5’ TCGACGCTCCCAAGGACTTC
SCO5570 real time-3’ ACGAGCACCCCTTCCATGAC

SCO5571 real time-5’ CACCGCCGGTCGCAGTGGAA
SCO5571 real time-3’ AAGTGCCGCAAGCCGGGCAC

SCO5572 real time-5' GTCCCCAAGAAGGCGGAAGA
SCO5572 real time-3' GCGTAGGAACGGTGGGTCAG

SCO6685 real time -5’ GGGGGAGATGGTTCGTATTG
SCO6685 real time -3’ AGGCACTCTCCGTCCACGAC

T7-SCO6685-ORF-5’ TAATACGACTCACTATAGGGACAAGGGGTGGGGGAGATGGTTCG
T7-SCO6685-ORF-3’ CGGGACCTGACGGGACACTGGAAC

T7-SCO6685-ORF+UTR-5’ TAATACGACTCACTATAGGGGGCGAATTGCCTCCGATGCGGTTC
T7-SCO6685-ORF+UTR-3’ GGCATACGGGCGCAGAGCGGAACG

SCO2792-2793 RT -5’ GCAAGGAGATCGTCGCGGTGCTGC
SCO2792-2793 RT-3’ ATACGACGTGCTTCGCGGCGATCG

hrdB real time-5’ CAAGAAGGCGACGGCGAAGA
hrdB real time-3’ CCTCGTCCTCGTCGGACAGC

hrdB Northern probe -5’ CAAGAAGGCGACGGCGAAGA
hrdB Northern probe -3’ CCTCGTCCTCGTCGGACAGC

rRNA real time-5’ CCTGATGCCGAGCCGATTGT
rRNA real time-3’ GGGGCATTTTGCCGAGTTCC

Supplementary Material Legend

Fig. S1. Quantification of spore generation by J1501 and C120 (*absB* mutant) strains. (A) 10^5 spores were inoculated on rich R5 medium (three repeats for each strain). After 6 days of growth, all the spores generated were collected by scraping the surface of the plates, suspended in 90 ml H2O, and the OD_{450} value was detected. (B) 200 μL of spore suspension from each plate was diluted 10^5 times and inoculated on R5 medium. Resulting colonies were counted after 2 days.

Fig. S2. Treatment of *sti1*, *ramR*, and *absB* locus transcripts with purified AbsB protein *in vitro*. (A) DNA corresponding to *sti1* open reading frame (*sti1* ORF), or both the ORF and UTR regions (*sti1* ORF+UTR1 and *sti1* ORF+UTR 2) was incubated with purified wild type AbsB or mutant AbsB protein respectively, as described in the Experimental procedures. (B) *In vitro* cleavage of *absB* locus transcripts by AbsB protein (as positive control) (Xu et al., 2008). (C) *In vitro* cleavage of *ramR* transcripts by AbsB protein.

Fig. S3. Growth curves for J1501 (morphologically wild type), C120 (*absB* mutant), J-2792 (Δ*adpA*), C-2792 (*absB*, Δ*adpA*) strains on solid R5 medium.

Fig. S4. (A) RT-PCR analysis of *adpA-ornA* transcript in J1501 (morphologically wild type) and C120 (*absB* mutant) strains grown on R5 plates for 30 hours to 120 hours as indicated. *HrdB* transcript abundance was used as the positive control. Negative control RT-PCR reaction was performed without the addition of the reverse transcriptase. (B) Quantitative RT-PCR analysis of the mRNA abundance of *ornA* gene (SCO2793) in

J1501 (morphologically wild type), J-2792 (Δ*adpA*), C120 (*absB* mutant), and C-2792 (*absB*, Δ*adpA*) strains of *S. coelicolor* grown on R5 medium for 30 hours to 120 hours as indicated.

Table S1. Genes containing putative AdpA binding sites. The AdpA binding motif [TGGC(G/C)(G/A/T/C/null)G(A/T)(A/T) identified in *S. griseus*] was detected in *S. coelicolor* genes whose expression level increased or decreased > 6 fold by *absB* mutation (Huang et al., 2005). The sequences examined extend 300 nt in region 5' to the gene's open reading frame, or if the length of the 5' region is less than 300 nt, the intergenic region between two genes. Sequences 5' to the first gene in multi-gene operons were used for all the genes in the operon. We used a Perl script pattern match algorithm to find matches showing 100% identity with the probe sequence. (SCOID: the SCO number of the gene; Motif: the sequence of the AdpA binding motif found in the promoter region; Location: the position of the AdpA biding motif relative to the start codon of the open reading frame or the first open reading frame in the operon; Direction: the direction of the AdpA binding motif relative to the direction of the open reading frame).

Table S2. Putative AdpA binding motifs (TGGC(G/C)(G/A/T/C/null)G(A/T)(A/T) in all *S. coelicolor* genes. The search criteria used are described in Table S1.

Table. S3. Primers used in the experiments reported in this paper.
